# Mindfulness-Based Cognitive Therapy in Major Depressive Disorder: A Study Protocol of a Randomized Control Trial and a Case–Control Study With Electroencephalogram

**DOI:** 10.3389/fpsyt.2021.499633

**Published:** 2021-07-26

**Authors:** Tianran Zhang, Lanlan Wang, Yanle Bai, Wenqing Zhao, Yanru Wu, Wenhui Jiang, Qing Fan, Jianyin Qiu

**Affiliations:** Shanghai Mental Health Center, School of Medicine, Shanghai Jiao Tong University, Shanghai, China

**Keywords:** major depressive disorder, mindfulness based cognitive therapy, randomized-control trial, case-control study, electroencephalogram

## Abstract

**Background:** In this report, a study protocol for a randomized control trial is presented in an attempt to explore the efficacy of mindfulness-based cognitive therapy (MBCT) for major depressive disorder (MMD), and a case–control study is conducted to find the difference in electroencephalograms (EEGs) between MMD and normal controls.

**Design:** Seventy Chinese patients with MMD will be chosen for random division in the MBCT group or medication group, with half of the participants receiving common medication treatment [selective serotonin reuptake inhibitors (SSRIs)] and half receiving MBCT as a supplement to the common medication treatment. All participants, namely, 70 MMD cases and 35 matched normal controls, will be tested by a range of scales and EEG at baseline (week 0), mid-intervention (weeks 2, 4, and 6), post-intervention (week 8), and 6-months follow-up (weeks 12, 20, and 32).

**Discussion:** The findings of this study will provide a positive reference for the treatment of depression and future research on MBCT treatment mechanism.

**Trial Registration:** NCT03558256. Registered: June 13, 2018—retrospectively registered, https://clinicaltrials.gov/ct2/show/NCT03558256.

## Background

Major depressive disorder (MDD) is a common mental disorder, which is characterized by high incidence, high recurrence, high disability, and high suicide rate. The prevalence rate of MDD in the general population is ~10%, while the lifetime prevalence rate reaches up to 16% (1). As one leading cause of disability worldwide (2), MDD affects not only psychological and biological capacity but also quality of life and social function. Except for the heavy burden of this disease, there is a significant risk of relapse and recurrence for people suffering from MDD. It is about 50% after one depressive episode, increasing to 70% after two episodes, and up to 90% after three episodes (3).

It has been demonstrated in clinical research that about 30–40% of patients with depression who have received first-line antidepressant treatment fail to respond to it regardless of the dose and duration (4). Except for medication and physical therapy, psychotherapy, especially cognitive behavior therapy (CBT), has been a significant clinical treatment method for patients with depression for a long term. However, even after having received multiple treatments, such as medications and/or psychotherapy, the majority of patients with MDD fail to recover completely from it and still present with depressive symptoms (5). Therefore, it is an important task and also a challenge to explore optimal treatment methods for MDD.

Mindfulness-based cognitive therapy (MBCT) is an evidence-based psychotherapy, which was developed by Segal et al. (6) and specifically designed to prevent the relapse and recurrence of depression. The form of MBCT is an 8-weeks concentrated group intervention with systematic training and daily homework between each session. Through integrating mindfulness with the elements of CBT for depression, MBCT is beneficial to MDD patients seeking an inner experience and developing a “distanced” and “decentered” perspective. Mindfulness derives from the East and has been generally defined as “the awareness that emerges through paying attention to purpose, in the present moment, and non-judgmentally to the unfolding of experience moment by moment” (7). The focus of the structured mindfulness meditation training of MBCT is placed on the mindfulness-based stress reduction (MBSR) program. According to it, patients are taught to become more aware of their feelings, body sensations, and thoughts and establish a new relationship with themselves through several practices, such as body scanning, mindfulness breathing, mindfulness meditations, and so on. In addition, the cognitive theoretical basis of MBCT is that depression is associated with the distorted automatic modes of feelings (distrusting objective facts), thoughts (self-critical rumination), and behaviors (avoidance) (8), which is based on the theory of CBT. MBCT is intended to enable patients to learn the “doing” mode instead of the “automatic pilot” mode and develop constructive actions in order to relieve themselves from feelings of distress.

Since the appearance of MBCT, there are accumulating empirical studies about the efficacy of MBCT for MDD. As per the findings in some studies, MBCT can reduce the risk of MDD relapse/recurrence and relieve residual symptoms (9). According to a meta-analysis, the risk ratio for MBCT is 0.66 compared with treatment as usual (TAU), related to 34% of risk reduction. Besides, in some studies, the effectiveness of MBCT is equal to antidepressant medication for the prevention of relapse in MDD (10). Godfrin et al. (11) maintain that MBCT plus TAU can improve the mood state and life quality of patients with the recurrence of depressive episodes in both short and long terms. For non-melancholic depression, Manicavasgar et al. believe that MBCT is as effective as CBT (12). Although there are abundant studies on MBCT for MDD, randomized control trials (RCTs) are not employed in some of them (13, 14), and some only focus on the exploration of the short-term effect (15) or residual symptoms (16). Moreover, there are few studies on this field in China. Therefore, the efficacy of MBCT in the treatment of depression in China with RCT will be explored from a long-term perspective.

At present, the brain mechanism of MBCT in the treatment of depression is still unclear. In the study of EEG, event-related potential P300 and error-related negativity (ERN) have attracted attention. It has been shown in some studies that depression presents with a decrease of amplitude in patients with P300 delay latency, which suggests that patients with depression have cognitive deficits. In another study, it has been demonstrated that mindfulness can improve the normal individual's attentional quality, enhance the ability to resist distraction stimulation, and reduce the distraction-induced P3a amplitude. In addition, it has been proven in a study of normal subjects (17) that after 3 months of mindfulness training, individuals would pay less attention to instantaneous detachment, which indicates that mindfulness allows individuals to be constantly alert to different stimuli. Therefore, it can be hypothesized that MBCT can improve the attentional quality of patients with depression, make them pay more attention to resource allocation, and help them improve the abnormality of P300.

According to some studies, the amplitude of ERN and negative emotions would increase in patients with moderate and mild depression; severe depressive patients with reduced or no changes in ERN amplitude may be caused by emotional apathy and a lack of interest. Abnormalities in ERN suggest a defect in the response monitoring in patients with depression, which is a manifestation of cognitive decline. Following ERN, there is a popularity for the study of non-meditators for error positivity, and it has been found that mindfulness can reduce the average amplitude of Pe (error positivity). (18) conducted an investigation on the long-term effects of mindfulness meditation on ERN and Pe, and they found that the amplitude of ERN is positively correlated with meditation exercises and the acceptance of emotions. Based on the above studies, it can be hypothesized that MBCT may improve the ability of patients to monitor their own emotional receptivity and response, thus improving the abnormality of ERN. Therefore, in this study, a comparison will be drawn on the changes of resting EEG, P300, and ERN in patients with depression before and after MBCT and the differences between MDD and normal controls, with the aim of exploring the efficacy and mechanism of MBCT treatment.

The purpose of this study is to explore whether medication plus MBCT treatment is more effective compared with common medication treatment in patients with depression, and the relationship between the variation of P300 and ERN in MDD and clinical depressive symptoms and these EEG changes after MBCT treatment. Hence, an exploration will be performed on the correlation between the control group, MDD patients, and the MDD patients intervened by MBCT. There are several hypotheses proposed in this paper, namely, that (1) medication plus MBCT treatment will be superior to medication alone in MDD after 8 weeks of intervention and 6-months follow-up; (2) the variation of P300 and ERN in MDD is related to depressive symptoms; and (3) MBCT treatment will change the EEG of MDD patients.

## Methods/Design

### Study Design

**Figure 1:**
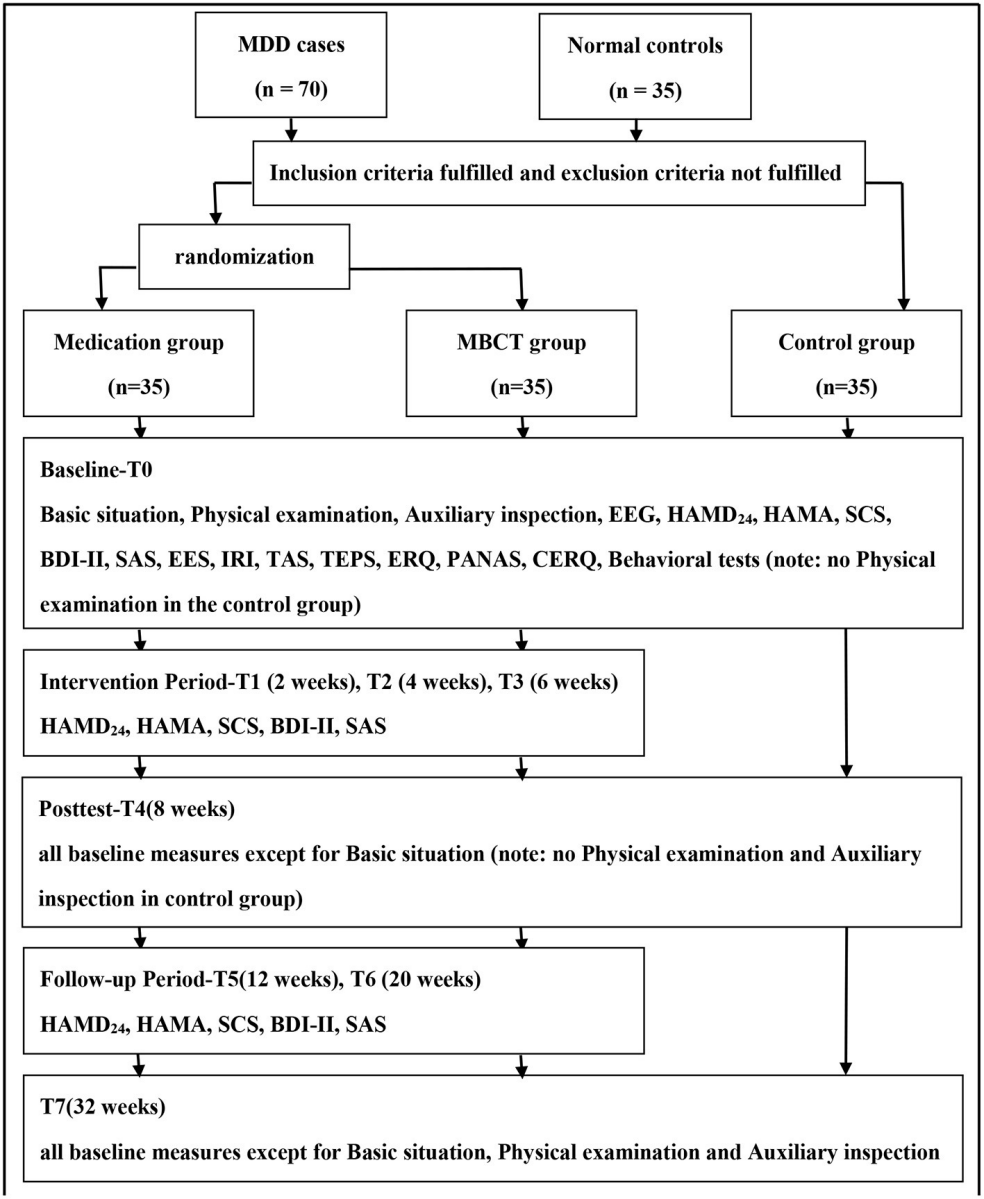
Study design and measurement time points. EEG, electroencephalogram; HAMD24, Hamilton Depression Scale-24; HAMA, Hamilton Anxiety Scale; SCS, Self-Compassion Scale; BDI-II, Beck Depression Inventory-II; SAS, Self-rating Anxiety Scale; EES, Emotional Expressivity Scale; IRI, Interpersonal Reactivity Index; TAS, Toronto Alexithymia Scale; TEPS, Temporal Experience of Pleasure Scale; ERQ, Emotion Regulation Questionnaire; PANAS, Positive Affect and Negative Affect Scale, CERQ, Cognitive Emotion Regulation Questionnaire.

As shown in Figure 1, the study is designed as a prospective, assessor-blinded, randomized control, case–control clinical trial with 70 MDD cases and 35 matched normal controls. According to DSM-IV criteria, all MDD cases will be randomized to the MBCT group or medication group through a predetermined random table. Half of the participants will receive the common medication treatment [selective serotonin reuptake inhibitors (SSRIs)], approved by the China Food and Drug Administration (SFDA) for the treatment of depression (fluoxetine, paroxetine, fluvoxamine, sertraline, citalopram, and escitalopram), while the other half of the participants will receive MBCT as a supplement to the common medication treatment.

All participants will receive an electroencephalogram at baseline (T0), after the 8-weeks intervention (T4), and at the end of the study (T7), in a view to investigate the change of the resting state of the brain, P300 potential, and ERN potential in patients with depression before and after MBCT treatment. Meanwhile, behavioral tests will be conducted to evaluate all participants with the electroencephalogram, in an attempt to discover behavioral indicators of MDD symptoms and the efficacy of MBCT. Moreover, some psychological scales will be used at the point of T0, T4, and T7 to evaluate the primary outcome-clinical depression symptoms (as measured by HAMD_24_), secondary outcomes-anxiety (HAMA), self-compassion [Self-Compassion Scale (SCS)], and other possible indicators, such as capabilities related to emotions (Beck Depression Inventory-II, Self-rating Anxiety Scale, Emotional Expressivity Scale (EES), Toronto Alexithymia Scale (TAS), Temporal Experience of Pleasure Scale (TEPS), Emotion Regulation Questionnaire (ERQ), and Positive Affect and Negative Affect Scale), empathy (Interpersonal Reactivity Index), and cognition [Cognitive Emotion Regulation Questionnaire (CERQ)].

After signing the informed consent form, MDD patients will be divided into two groups randomly by the table generated by Microsoft Excel 2010, which provides a guarantee for the random distribution between both groups. Patients in each group will be allotted by the research coordinator so that evaluators are blind to it, and they will be required not to expose the intervention conditions to the evaluators. In addition to the same assessments with normal controls at T0, T4, and T7, all MDD cases will be evaluated every 2 weeks during the intervention, which indicates that they will receive assessments of HAMD24, HAMA, SCS, BDI-II, and SAS at the point of T1 (week 2), T2 (week 4), and T3 (week 6).

The medical follow-up for MDD cases will be carried out after an 8-weeks intervention. Participants in the medication group will continue their treatment methods without changing the types and doses of medication compared with what they use in the intervention period. Similarly, participants in the MBCT group will maintain their medication treatment and conduct MBCT exercises at home. The long-term efficacy will be evaluated by the same scales during the intervention, and at 1 month (T5) and 3 months (T6) after the intervention.

### Sample/Participants

#### Sample Size

A power analysis will be conducted by the program “G^*^Power.” Based on the former theoretical considerations and results of comparable studies, the priori test power will be assumed to be 0.8 (1–β) and an effect size of d will be assumed to be 0.8. The minimum sample size of n1 = n2 = 26 will be adopted to detect the effect through a *t*-test at a *p* < 0.05 level of significance. The dropout rate will be assumed to be ~20% and the clinical feasibility will also be considered, n1 = n2 = 33. By taking the randomization into account, the targeted total sample size will be expressed as n3 = 70. On the grounds that the normal controls are matched with patient sample cases at 0.5:1, the sample size of normal controls will be n4 = 35. Therefore, 105 participants will be included in this study, namely, 70 patients and 35 normal controls.

#### Inclusion and Exclusion Criteria

The MDD patients of this study will be selected from the psychological counseling clinic and psychiatry clinic in Shanghai Mental Health Center, while the normal controls will be selected through advertisements. The specific inclusion and exclusion criteria are as follows:

Inclusion criteria for patients:

1. Male or female, age between 18 and 55 years.
2. Junior/middle school education or above.
3. Meet the DSM-IV diagnosis of MDD by the evaluation of the Chinese version of M.I.N.I. and the clinical diagnosis of one associate chief and above psychiatrist.
4. *HAMD*_24_ score ≥8.
5. Have not yet accepted psychiatric medication, or had received irregular medication treatment and had discontinued it for 8 weeks.
6. Have enough visual and acoustic ability to complete the inspection required by this study.
7. Participate in this study on a voluntary basis; he/she or his/her guardian shall sign the informed consent form after understanding the nature of this study.

Inclusion criteria for normal controls:

1. Healthy population matched patients in age, gender, and education; male or female; age between 18 and 55 years; junior/middle school education or above.
2. Exclude the possibility of mental disorders by the evaluation of the Chinese version of M.I.N.I. and psychiatric interview of one associate chief and above psychiatrist.
3. *HAMD*_24_ score <8; HAMA score <7.
4. No psychotropic drug history.
5. No history of two lines of three generations of mental disorders.
6. Have enough visual and acoustic ability to complete the inspection required by this study.
7. Participate in this study on a voluntary basis; he/she or his/her guardian shall sign the informed consent form after understanding the nature of this study.

Exclusion criteria for patients:

1. Meet DSM-IV Axis I disorder diagnostic criteria for other psychiatric disorders.
2. Present with severe depression symptoms (HAMD24 score > 35), psychotic symptoms, negative self-concept, or a risk of suicide.
3. Suffering from severe physical disease or central nervous system disease, and with substance abuse.
4. Pregnant women or getting ready for being pregnant and lactating women.
5. Had previously received systematic MBCT intervention, with no significant effect.

Exclusion criteria for normal controls:

1. Have negative self-concept or a risk of suicide.
2. Suffer from severe physical disease or central nervous system disease, and with substance abuse.
3. Pregnant women or getting ready for being pregnant and lactating women.

### The Interventions

#### The Setting

The MBCT intervention will be composed of 8 weekly 2-h sessions with closed groups, each of which will be composed of two instructors and more than six patients. The intervention will be held in an outpatient setting at the Shanghai Mental Health Center in Shanghai Jiao Tong University School of Medicine. The instructors in this study will be trained and certificated psychiatrists and psychotherapists with rich working experience in MDD, and they will receive weekly supervision conducted by a senior supervisor.

#### The Medication Treatment

All patients in this study will receive the medication treatment. Both the MBCT group and the medication group can choose to employ SSRI drugs, which have been approved by the China Food and Drug Administration (SFDA) for the treatment of depression (fluoxetine, paroxetine, fluvoxamine, sertraline, citalopram, and escitalopram). The initial dose will conform to the drug instructions, the dosage can be adjusted once a week, and the maximum dosage cannot exceed the maximum amount by the instructions. MDD patients with sleep disorders can employ drugs combined with benzodiazepine drugs, with a duration of no more than 2 weeks; while other psychotropic drugs are not allowed. All drugs to be used in this study are common clinical drugs with good security. Common adverse reactions include nausea, dry mouth, constipation, diarrhea, indigestion, dizziness, drowsiness, fatigue, sweating, heart palpitations, delayed ejaculation in men, and occasionally increased blood aminotransferase without symptoms (19).

#### The MBCT Program

Different from the medication group, participants in the MBCT group will receive the MBCT intervention except for the common medication treatment. The intervention will be implemented by the manual translated from the MBCT for Depression (6). The modified version of the program has the same structure as the original manual combined with the mindfulness training and the cognitive elements from CBT, but the content is more suitable for Chinese speakers. Through the translation, plain words are adopted. More psycho-education will be performed at the beginning and end of the intervention for patients and family members to reduce their stigma.

To be specific, the themes of the eight sessions can be defined as “automatic guided,” “deal with obstacles,” “vipassana breathing,” “live here and now,” “let nature take its course,” “thoughts are not facts,” “how to take care of yourself,” and “use what you learn to deal with the future.” Except for training during these sessions, participants will conduct exercises in their homework as per the prescribed length of time to strengthen what they learn between sessions. Besides, the audio files of mindfulness practices in this program will be provided.

### Outcome Measures

Efficacy will be evaluated on the main basis of HAMD_24_. Due to the fact that MBCT not only works on depression symptoms but also on other emotional problems, such as anxiety, HAMA will also be employed to evaluate the efficacy. For HAMD_24_ and HAMA, the reduction score and rate will be the statistical index of depressive symptoms. More specifically, the reduction rate ≥50% represents an obvious effect, <50% and ≥25% represents a common effect, while <25% represents no clinic effect. Meanwhile, the score of the SCS will be regarded as the secondary outcome to evaluate the efficacy of the intervention. In addition, some psychological scales, behavioral tests, and electroencephalograms will also be adopted to assess the changes in emotion, cognition, and other characteristics.

#### Primary Outcome Measure

The Hamilton Depression Scale-24 (HAMD-24) (20) is the most common other-rating scale, which can be employed to measure the severity of depression symptoms for adults. It is a clinical instrument with favorable validity and reliability, with the majority of 24 items weighted from 0 to 4.

#### Secondary Outcome Measures

The Hamilton Anxiety Scale (HAMA) (21) is a common clinician-administered scale, which can be employed to measure the state of anxiety among adults with anxiety symptoms. There are 14 items on a five-point rating scale.

It has been demonstrated that the SCS (22) is an instrument with favorable reliability and validity. There are 26 items that can be divided into six subscales (out of five points from almost never to almost always), namely, self-kindness, self-judgment, common humanity, isolation, mindfulness, and over-identification.

#### Other Psychological Scales

The EES (23) is a 17-item self-report six-point Likert-type scale, which is designed to evaluate the extent to which people outwardly display their emotions. On the grounds that the Chinese version of the EES features high internal consistency and test–retest reliability, it will be employed in this study.

The TAS (24) is a 20-item self-report instrument with each item rated from 1 (strongly disagree) to 5 (strongly agree), which is designed to measure degrees of alexithymia. It consists of three factors, namely, difficulty identifying, difficulty describing, and an externally oriented cognitive style of thinking.

The TEPS (25) comprises 20 items, and it is designed to evaluate the anticipatory and consummatory facets of pleasure. In this study, the Chinese version of TEPS rated from 1 (very false for me) to 6 (very true for me) will be employed.

The ERQ (26) is a 10-item seven-point measure. In this study, the Chinese version of it will be employed to survey the individual reappraisal and inhibitive expression strategies.

The CERQ (27) comprises 28 items, and it is designed to evaluate specific cognitive emotion regulation strategies. There are nine factors of CERQ, namely, self-blame, acceptance, rumination, positive refocusing, refocusing on planning, positive reappraisal, putting into perspective, catastrophizing, and other-blame.

#### Electroencephalogram

In this study, an exploration will be conducted on the change of the resting state of the brain, P300 potential, and ERN potential in patients with depression before and after MBCT treatment.

#### Behavioral Test

The behavioral tests include two computer experiments, which will be employed to investigate the difference of emotional mechanism between MDD cases and normal controls. One is the emotional Stroop task, and the other is an emotional regulation experiment.

### Data and Statistical Analyses

In this study, the ANOVA test will be performed for comparisons at baseline with the analysis of variance for the continuous variables; while the chi-square test will be adopted for the categorical variables. In the primary analysis, independent sample *t*-test will be applied for a comparison of the differences in treatment outcomes between both groups; while the comparisons at weeks 2, 4, and 6 will be conducted by multiple testing. A two-sided *p* < 0.05 will be considered as statistically significant. It is the same condition for the secondary analysis. The corresponding 95% CIs will be calculated whenever possible. In the secondary analysis, continuous outcomes from baseline to week 8 will be analyzed with general linear mixed models. The main predictors of interest including time, treatment group, and interaction between them, with age, gender, education level, and baseline score will be entered as covariates and treatment site as a random effect in the model. Restricted maximum likelihood will be adopted in the mixed models. Besides, the above analyses will be repeated after supplementing 1-month, 3-months, and 6-months follow-up scores to assess the maintenance of treatment.

## Discussion

So far, research about the efficacy of MBCT for depression has been very rich. Different from previous studies, the focus of this study will be placed on patients with depression who are currently untreated, but not in remission or relapse. For patients with exacerbated depression, medication therapy and CBT are relatively mature treatments. However, as mentioned above, there are still many patients with depression who cannot benefit from it, with a tendency to relapse. Therefore, it is still necessary to explore an optimal treatment for depression. It has been shown in many studies that MBCT can reduce the recurrence of depression and relieve depressive symptoms effectively. For that reason, an exploration will be conducted on whether MBCT combined with traditional medicine is beneficial for more patients with depression.

As far as we know, there is no research on the brain mechanism of MBCT in the treatment of depression by EEG. Therefore, this is the first large sample randomized controlled study in this field. Based on studies about the brain mechanism of depression and the change after mindfulness training on the brain, it can be assumed that MBCT can improve depressive patients with intentional attention ability, allocate attentional resources more reasonably, and improve abnormal P300. Besides, it can be assumed that MBCT is beneficial to patients enhancing their own emotional acceptance and reaction monitoring in order to improve the ability of abnormal ERN. It is expected that the results of this study will provide a novel understanding of the brain mechanism of MBCT in the treatment of depression.

Moreover, as a psychological therapy that integrates Eastern and Western cultures, MBCT can be more acceptable to Chinese patients. Therefore, this treatment method in China may be more popular than traditional CBT treatment. It has been shown in some studies that MBCT is not only effective for symptom improvement but also important for the improvement of life quality and social function. Therefore, this study will be continued for 6 months to explore the long-term efficacy of the treatment method and the improvement of life quality and social function after the treatment.

Until now, some patients have been recruited for the pilot study to prove the rationality and feasibility of the proposed study. Besides, the formal study formulated in this paper has not initiated the patient recruitment at the time of submission.

If the findings of this study are in line with its assumptions, there will be favorable treatment options for patients with depression. Due to the fact that MBCT is a means of group therapy and is easy to be implemented for a long time, it contributes to the treatment of more patients with depression each time and enables them to consolidate the improvement after the treatment. Therefore, this is a cost-effective treatment and easy to be implemented. In general, the findings of this study will exert a favorable impact on the treatment of depression and future studies on the treatment mechanism of MBCT. Based on this study, the focus of further studies shall be placed on clarifying the treatment mechanism of MBCT, such as psychological mechanism and brain mechanism, and improvement in the treatment of depression.

## Trial Status

The protocol version number is 02 and the date is April 11, 2016. The recruitment will begin in March 2019, and the study is expected to last 2 years so that the approximate date when recruitment will be completed should be in March 2021.

## Ethics Statement

The studies involving human participants were reviewed and approved by Ethics committee of the Shanghai Mental Health Center. The patients/participants provided their written informed consent to participate in this study.

## Author Contributions

All authors listed have made a substantial, direct and intellectual contribution to the work, and approved it for publication.

## Conflict of Interest

The authors declare that the research was conducted in the absence of any commercial or financial relationships that could be construed as a potential conflict of interest.

## Publisher's Note

All claims expressed in this article are solely those of the authors and do not necessarily represent those of their affiliated organizations, or those of the publisher, the editors and the reviewers. Any product that may be evaluated in this article, or claim that may be made by its manufacturer, is not guaranteed or endorsed by the publisher.

## References

[1] Kessler RC Aguilar-Gaxiola S Alonso J Chatterji S Lee S Ormel J. The global burden of mental disorders: an update from the WHO World Mental Health (WMH) Surveys. Epidemiol Psich Soc. (2009) 18:23–33. 10.1017/S1121189X00001421 19378696 PMC3039289

[2] World Health Organization. Depression Fact Sheet. (2012). Retrieved from: http://www.who.int/mediacentre/factsheets/fs369/en/March2014

[3] Kessing LV Hansen MG Kessing LV Hansen GM Andersen G Angst J. The predictive effect of episodes on the risk of recurrence in depressive and bipolar disorders—A life-long perspective. Acta Psychiatr Scand. (2004) 109:339–44. 10.1046/j.1600-0447.2003.00266.x 15049770

[4] Bloom FE Kupfer DJ Bunney BS. Psychopharmacology: the Fourth Generation of Progress. New York, NY: Raven Press (1995).

[5] Pigott HE Leventhal AM Alter GS Boren JJ. Efficacy and effectiveness of antidepressants: current status of research. Psychother Psych. (2010) 79:267–79. 10.1159/000318293 20616621

[6] Segal ZV Williams JMG Teasdale JD. Mindfulness-Based Cognitive Therapy for Depression: A New Approach to Preventing Relapse. New York, NY: Guilford Press (2002).

[7] Kabat-Zinn J. Mindfulness-based interventions in context: past, present, and future. Clin Psych Sci Practice. (2003) 10:144–56. 10.1093/clipsy.bpg016

[8] Lau MA Segal ZV Williams JMG. Teasdale's differential activation hypothesis: implications for mechanisms of depressive relapse and suicidal behaviour. Behav Res Ther. (2004) 42:1001–17. 10.1016/j.brat.2004.03.003 15325898

[9] Zhang Z Zhang L Zhang G Jin J Zheng Z. The effect of cbt and its modifications for relapse prevention in major depressive disorder: a systematic review and meta-analysis. BMC Psychiatry. (2018) 18:50. 10.1186/s12888-018-1610-5 29475431 PMC6389220

[10] Piet J Hougaard E. The effect of mindfulness-based cognitive therapy for prevention of relapse in recurrent major depressive disorder: a systematic review and meta-analysis. Clin Psych Rev. (2011) 31:1032–40. 10.1016/j.cpr.2011.05.002 21802618

[11] Godfrin KA Van Heeringen C. The effects of mindfulness-based cognitive therapy on recurrence of depressive episodes, mental health and quality of life: a randomized controlled study. Behav Res Ther. (2010) 48:738–46. 10.1016/j.brat.2010.04.006 20462570

[12] Manicavasgar V Parker G Perich T. Mindfulness-based cognitive therapy vs cognitive behaviour therapy as a treatment for non-melancholic depression. J Affect Disord. (2011) 130:138–144. 10.1016/j.jad.2010.09.027 21093925

[13] Kenny MA Williams JMG. Treatment-resistant depressed patients show a good response to mindfulness-based cognitive therapy. Behav Res Ther. (2007) 45:617–25. 10.1016/j.brat.2006.04.008 16797486 PMC2808477

[14] Eisendrath SJ Delucchi K Bitner R Fenimore P Smit M McLane M. Mindfulness-based cognitive therapy for treatment-resistant depression: a pilot study. Psychother Psychos. (2008) 77:319–20. 10.1159/000142525 18600038

[15] Crane C Barnhofer T Duggan DS Hepburn S Fennell MV Williams JMG. Mindfulness-based cognitive therapy and self-discrepancy in recovered depressed patients with a history of depression and suicidality. Cogn Therap Res. (2008) 32:775. 10.1007/s10608-008-9193-y

[16] Kingston T Dooley B Bates A Lawlor E Malone K. Mindfulness-based cognitive therapy for residual depressive symptoms. Psych Psychother Theory Res Pract. (2007) 80:193–203. 10.1348/147608306X116016 17535594

[17] Slagter HA Lutz A Greischar LL Francis AD Nieuwenhuis S Davis JM. Mental training affects distribution of limited brain resources. PLoS Biol. (2012) 5:e138. 10.1371/journal.pbio.0050138 17488185 PMC1865565

[18] Rimma T Michael I. Meditation, mindfulness and executive control: the importance of emotional acceptance and brain-based performance monitoring. Soc Cogn Affect Neurosci. (2013) 8:85–92. 10.1093/scan/nss045 22507824 PMC3541488

[19] Procyshyn RM Bezchlibnyk-Butler KZ Jeffries JJ. (Eds.). Clinical Handbook of Psychotropic Drugs. Cambridge: Hogrefe Publishing. (2017). 10.1027/00496-000

[20] Zimmerman M Martinez JH Young D Chelminski I Dalrymple K. Severity classification on the hamilton depression rating scale. J Affect Disorder. (2013) 150:384–8. 10.1016/j.jad.2013.04.028 23759278

[21] Zimmerman M Martin J Clark H McGonigal P Harris L Holst CG. Measuring anxiety in depressed patients: a comparison of the Hamilton anxiety rating scale and the DSM-5 Anxious Distress Specifier Interview. J Psychiatr Res. (2017) 93:59–63. 10.1016/j.jpsychires.2017.05.014 28586699

[22] Neff KD. The development and validation of a scale to measure self-compassion. Self Identity. (2003) 2:223–50. 10.1080/15298860309027

[23] Chan RC Wang Y Li H Shi Y Wang Y Liu W. A 2-stage factor analysis of the Emotional Expressivity Scale in the Chinese context. Psychologia. (2010) 53:44–50. 10.2117/psysoc.2010.44

[24] Zhu X Yi J Yao S Ryder AG Taylor GJ Bagby RM. Cross-cultural validation of a Chinese translation of the 20-item Toronto Alexithymia Scale. Compreh Psych. (2007) 48:489–96. 10.1016/j.comppsych.2007.04.007 17707259

[25] Chan RC Shi YF Lai MK Wang YN Wang Y Kring AM. The Temporal Experience of Pleasure Scale (TEPS): exploration and confirmation of factor structure in a healthy Chinese sample. PLoS ONE. (2012) 7:e35352. 10.1371/journal.pone.0035352 22530007 PMC3329425

[26] Li WANG. Hengchao LIU Zhongquan L. Reliability and validity of emotion regulation questionnaire Chinese revised version. Chin J Health Psychol. (2007) 6:013. 10.3969/j.issn.1005-1252.2007.06.034

[27] Zhu X Auerbach RP Yao S Abela JR Xiao J Tong X. Psychometric properties of the cognitive emotion regulation questionnaire: chinese version. Cogn Emot. (2008) 22:288–07. 10.1080/0269993070136903526925586

